# Endocrinology and the Lung: Exploring the Bidirectional Axis and Future Directions

**DOI:** 10.3390/jcm14196985

**Published:** 2025-10-02

**Authors:** Pedro Iglesias

**Affiliations:** 1 Department of Endocrinology and Nutrition, Hospital Universitario Puerta de Hierro Majadahonda, C. Joaquín Rodrigo, 1, 28222 Majadahonda, Madrid, Spain; piglo65@gmail.com; 2 Instituto de Investigación Sanitaria Puerta de Hierro Segovia de Arana, 28222 Majadahonda, Madrid, Spain

**Keywords:** endocrinology, pulmonary function, neuroendocrine tumors, adrenocorticotropic hormone (ACTH), thyroid hormones, carcinoid, serotonin, sleep apnea syndromes, paraneoplastic syndromes

## Abstract

The lung is increasingly recognized as an organ with dual endocrine and respiratory roles, participating in a complex bidirectional crosstalk with systemic hormones and local/paracrine activity. Endocrine and paracrine pathways regulate lung development, ventilation, immunity, and repair, while pulmonary cells express hormone receptors and secrete mediators with both local and systemic effects, defining the concept of the “endocrine lung”. This narrative review summarizes current evidence on the endocrine–pulmonary axis. Thyroid hormones, glucocorticoids, sex steroids, and metabolic hormones (e.g., insulin, leptin, adiponectin) critically influence alveologenesis, surfactant production, ventilatory drive, airway mechanics, and immune responses. Conversely, the lung produces mediators such as serotonin, calcitonin gene-related peptide, endothelin-1, leptin, and keratinocyte growth factor, which regulate vascular tone, alveolar homeostasis, and immune modulation. We also describe the respiratory manifestations of major endocrine diseases, including obstructive sleep apnea and lung volume alterations in acromegaly, immunosuppression and myopathy in Cushing’s syndrome, hypoventilation in hypothyroidism, restrictive “diabetic lung”, and obesity-related phenotypes. In parallel, chronic pulmonary diseases such as chronic obstructive pulmonary disease, interstitial lung disease, and sleep apnea profoundly affect endocrine axes, promoting insulin resistance, hypogonadism, GH/IGF-1 suppression, and bone metabolism alterations. Pulmonary neuroendocrine tumors further highlight the interface, frequently presenting with paraneoplastic endocrine syndromes. Finally, therapeutic interactions are discussed, including the risks of hypothalamic–pituitary–adrenal axis suppression with inhaled corticosteroids, immunotherapy-induced endocrinopathies, and inhaled insulin. Future perspectives emphasize mapping pulmonary hormone networks, endocrine phenotyping of chronic respiratory diseases, and developing hormone-based interventions.

## 1. Introduction

The significance of the endocrine–pulmonary axis lies in redefining the lung, not only as a site of gas exchange but also as a hormonally responsive and producing organ.

Systemic hormones play a pivotal role in pulmonary development and function. Thyroid hormones are essential for alveolar maturation and surfactant synthesis [1], while glucocorticoids drive fetal lung maturation and support antenatal steroid therapy [2,3,4]. Sex steroids influence bronchial tone, ventilatory drive, and inflammatory responses [5,6,7,8,9,10,11], contributing to sex differences in asthma and chronic obstructive pulmonary disease (COPD) [12,13]. Metabolic hormones such as insulin, leptin, and IGF-1 also modulate pulmonary mechanics and immune function. Consequently, endocrine disorders including hypothyroidism [14], diabetes mellitus [15,16], acromegaly [15,17], and obesity [18] often manifest with pulmonary alterations, ranging from hypoventilation to obstructive sleep apnea.

Conversely, pulmonary diseases exert systemic endocrine effects. COPD, interstitial lung disease, and obstructive sleep apnea are associated with insulin resistance [19], hypogonadism [20], osteoporosis [21], and hypothalamic–pituitary–adrenal axis [22] dysregulation. These sequelae increase morbidity and complicate clinical management, underscoring the bidirectional nature of endocrine–pulmonary interactions.

Beyond being hormone targets, the lungs also display intrinsic endocrine activity. Pulmonary neuroendocrine cells (PNECs) are specialized epithelial sensors that release neuropeptides and hormones in response to environmental stimuli, linking respiratory, neural, and endocrine systems [23]. Moreover, pulmonary neuroendocrine tumors and small-cell lung carcinoma secrete ectopic hormones, producing paraneoplastic syndromes such as syndrome of inappropriate antidiuretic hormone secretion (SIADH) and ectopic Cushing’s syndrome [24].

The relationship between endocrinology and pulmonology has been recognized for decades, though traditionally studied in isolation. Early work identified the crucial role of thyroid hormones and glucocorticoids in fetal lung development [2,4]; Later, the association of acromegaly and obesity with sleep apnea [20,25], as well as the description of paraneoplastic syndromes in small-cell lung carcinoma [24]. These findings consolidated the dual concept of the lung as both hormone target and source, but integration into a unified framework remains limited.

However, significant knowledge gaps persist. The mechanisms through which metabolic hormones (insulin, leptin, adipokines) and sex steroids influence pulmonary physiology remain incompletely understood. The systemic endocrine consequences of chronic lung diseases are frequently underdiagnosed and undertreated, despite their impact on prognosis and quality of life. Moreover, the clinical significance of pulmonary neuroendocrine signaling is not yet fully appreciated in everyday practice, leaving an unmet need for translational research and interdisciplinary management.

Taken together, current evidence supports the concept that the lung is both an endocrine target and source, regulated by multiple hormonal axes with implications for pathophysiology, clinical care, and therapeutic innovation. Recognizing these interactions is essential for the early detection of systemic complications, the optimization of multidisciplinary management, and the identification of new therapeutic targets.

Accordingly, this narrative review is structured into four main sections. First, we address the physiological pathways of dual crosstalk between the endocrine and pulmonary systems. Second, we describe endocrine ailments with pulmonary involvement. Third, we examine lung conditions with endocrine manifestations, including subsections on pulmonary cancer/neuroendocrine tumors and non-cancerous lung conditions. Finally, we discuss therapeutic interferences, with emphasis on how pharmacological and interventional treatments affect both systems and how these insights may inform future interdisciplinary research.

## 2. Methods

The Medical Subject Headings (MeSH) terms used for the search included “endocrinology”, “lung”, “respiratory epithelium”, “pulmonary neuroendocrine cells”, “hormone receptors”, “thyroid hormones”, “glucocorticoids”, “sex steroids”, “insulin”, “IGF-1”, “leptin”, “adiponectin”, “endothelin-1”, and “paraneoplastic syndromes”. These terms were applied to PubMed/MEDLINE, the Cochrane Database of Systematic Reviews, and Embase.

The search focused on the most relevant English-language articles published within the last 10 years (up to 31 August 2025), although older publications of particular relevance were also considered.

Inclusion criteria comprised original studies, narrative and systematic reviews, and meta-analyses evaluating the relationship between endocrine pathways and pulmonary development, physiology, or disease. Exclusion criteria were abstracts, conference proceedings, and non-English publications.

## 3. Physiological Pathways of Dual Crosstalk

The lung is not merely a passive target of systemic hormones but functions as an active endocrine organ. Hormonal pathways regulate lung development, ventilation, immunity, and repair, while pulmonary cells express specific hormone receptors and secrete mediators with both local and systemic effects. This reciprocal interaction underpins the concept of the *“endocrine lung”*, linking hormonal regulation to respiratory health and disease.

### 3.1. Hormonal Control of Lung Development

**Figure 1:**
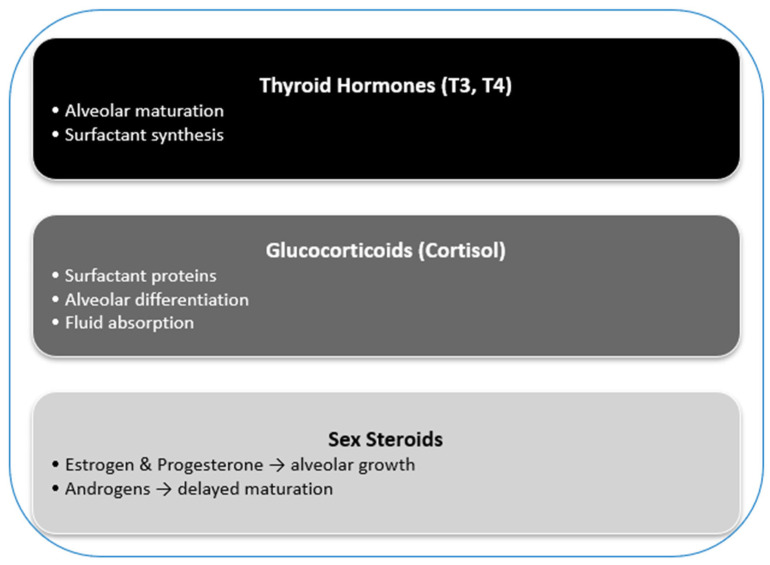
Hormonal control of lung development [1,2,3,4,5,6,7].

Hormonal pathways play a key role in pulmonar development (Figure 1).

Thyroid hormones (triiodothyronine, T3 and thyroxine, T4) are essential for alveolar maturation and surfactant production. In cultured fetal rabbit lung, T3 stimulates phosphatidylcholine synthesis, the major surfactant phospholipid, and acts synergistically with glucocorticoids [26]. In human lung explants (15–24 weeks), treatment with T3 and dexamethasone markedly increases choline incorporation into phosphatidylcholine and promotes lamellar body formation [27]. In murine models, prenatal hypothyroidism reduces alveolar septation and sustains elevated surfactant protein mRNA levels postnatally [28]. In preterm infants, low circulating T3 and thyroid-stimulating hormone (TSH) levels correlate with increased risk of respiratory distress syndrome [29]. These findings underscore the critical role of thyroid hormones in lung development and highlight their potential contribution to the pathogenesis of neonatal respiratory morbidity.

Glucocorticoids are equally critical for fetal lung maturation. They stimulate surfactant protein and phospholipid synthesis, enhance epithelial differentiation, and facilitate lung fluid absorption [30]. Experimentally, cortisol infusion in pregnant ewes increases alveolar surface area, reduces interalveolar wall thickness, and raises alveolar number without impairing fetal growth [31]. Conversely, adrenalectomy prevents the prepartum cortisol surge and reduces lung fluid secretion and clearance [32]. Clinically, antenatal glucocorticoid therapy accelerates lung maturation and lowers the incidence of respiratory distress syndrome [33]. Together, experimental and clinical evidence confirm the decisive role of glucocorticoids in fetal lung development and prevention of neonatal complications.

Sex hormones exert divergent effects on alveologenesis. Estrogen, the only steroid generating sexual dimorphism in alveolar number and size, promotes fluid absorption and alveolar regeneration via ERα and ERβ receptors. Progesterone supports differentiation and limits proliferation, complementing estrogen’s actions. Conversely, androgens such as dihydrotestosterone (DHT) enhance fibroblast and alveolar epithelial proliferation but delay maturation, resulting in impaired alveolar differentiation and contributing to the higher vulnerability of preterm males to respiratory distress syndrome [33].

### 3.2. Hormonal Regulation of Respiratory Function

**Figure 2:**
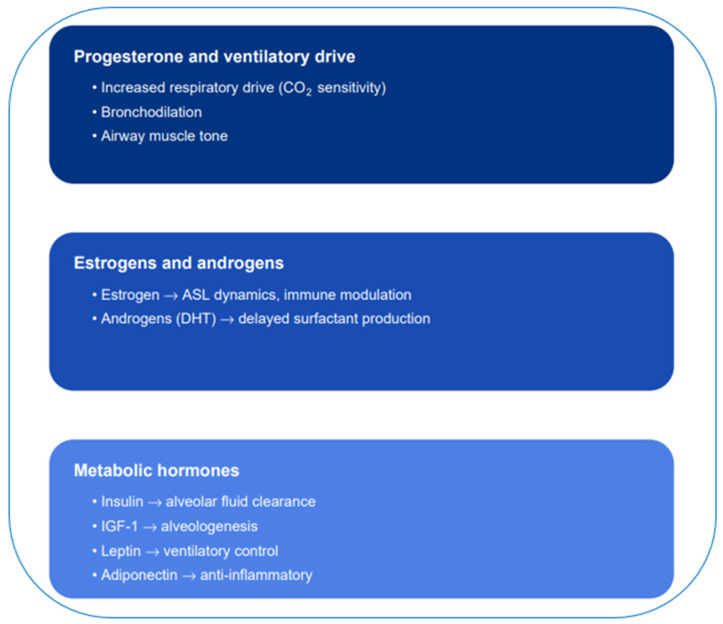
Hormonal regulation of respiratory function [8,9,10,11,12,13,14]. Abbreviations: ASL, Airway surface liquid; CO_2_, Carbon dioxide; DHT, Dihydrotestosterone, IGF-1, Insulin-like growth factor 1.

Reproductive and metabolic hormones exert a decisive influence on ventilation, lung mechanics and immunity (Figure 2).

Progesterone acts as a potent respiratory stimulant by enhancing central CO_2_ sensitivity, inducing bronchodilation, and increasing upper airway dilator muscle activity [34]. During pregnancy, elevated progesterone drives physiological hyperventilation characterized by increased tidal volume and reduced PaCO_2_ [35]. It also exerts protective effects against pharyngeal collapse and obstructive sleep apnea, although very high concentrations may promote nocturnal ventilatory instability [36,37,38]. After menopause, progesterone decline is linked to greater risk of sleep-disordered breathing [39,40]. Thus, progesterone emerges as a key modulator of ventilatory drive and female respiratory stability.

Estrogens and androgens modulate respiratory mechanics and immunity in opposing ways. Estrogen regulates airway surface liquid (ASL) dynamics, supports surfactant production, and shapes immune responses, thereby aggravating or alleviating diseases such as asthma, cystic fibrosis, and COVID-19 depending on the context [41]. Androgens, particularly dihydrotestosterone (DHT), delay surfactant production in fetal lungs, which contributes to the greater susceptibility of male neonates to respiratory distress syndrome [42].

Metabolic hormones also play critical roles in pulmonary physiology. Insulin enhances alveolar fluid clearance by upregulating epithelial sodium channel (ENaC) channels through the phosphatidylinositol 3-kinase/protein kinase B (PI3K/Akt) pathway and protects epithelial barrier integrity in pulmonary edema models [43]. IGF-1 supports postnatal lung growth and alveologenesis [44]. Leptin modulates central respiratory control, improves ventilatory responses to CO_2_, and prevents hypoventilation in animal models [45,46]. Adiponectin exerts anti-inflammatory effects, modulating immune tone in chronic respiratory diseases such as COPD [47].

### 3.3. Hormone Target Cells and Receptors in the Lung

The lung is not merely a passive target of systemic endocrine signals but expresses a broad network of hormone receptors in epithelium, endothelium and smooth muscle, allowing direct modulation of development, ventilation, repair, and immune tone (Table 1). This functional mapping highlights that systemic hormones exert not only indirect effects through metabolic or cardiovascular pathways but also direct, local actions on pulmonary physiology. Such mechanisms help explain why endocrinopathies such as hypothyroidism, hyperthyroidism, obesity, diabetes or Cushing’s syndrome alter respiratory mechanics and increase susceptibility to pulmonary complications, while at the same time opening new therapeutic opportunities targeting specific pulmonary hormone receptors.

#### 3.3.1. Thyroid Hormone Receptors (TRa, TRβ)

Type II pneumocytes express thyroid hormone receptors (TRα and TRβ), whose activation by triiodothyronine (T3) is essential for alveolar regeneration and differentiation into type I pneumocytes. T3 upregulates transcription factors such as KLF2 and CEBPA, preserves mitochondrial function, and prevents apoptosis. It also limits fibroblast activation and extracellular matrix production, protecting against fibrosis [48,49,50].

#### 3.3.2. Glucocorticoid Receptors (GRa, GRβ)

The glucocorticoid receptor (GR) is widely expressed in bronchial epithelium, endothelium, and smooth muscle, mediating anti-inflammatory effects [66,69,70]. The functional GRa isoform regulates gene transactivation and transrepression, inhibiting nuclear factor kappa-light-chain-enhancer of activated B cells (NF-κB) and activator protein 1 (AP-1), resolving inflammation, and reprogramming the transcriptome [71,72,73,74]. In contrast, GRβ, generated by alternative splicing and lacking binding capacity, acts as a dominant-negative inhibitor of GRa [51,71,75,76]. GRβ expression is upregulated in chronic inflammation under Th17 cytokines (IL-17A/F), raising the GRβ/GRa ratio and contributing to corticosteroid resistance, particularly in asthma and COPD [51,71,75,76].

#### 3.3.3. Estrogen and Progesterone Receptors (ERa, ERβ, PR)

Estrogen receptors are expressed in smooth muscle and epithelium, differentially modulating contractility and calcium signaling. ERβ reduces intracellular calcium and contractility, while ERa can potentiate contraction [5,10,52,53,77]. Estradiol acutely induces bronchodilation by inhibiting calcium entry, promoting sarcoplasmic reuptake, and activating cAMP/PKA [52,53,54,55]. At high concentrations, estradiol may paradoxically induce hyperreactivity by inhibiting Ca^2+^-ATPase [78]. In the ciliated epithelium, ERα36 increases after allergic sensitization [67,68]. Progesterone reduces ciliary beat frequency (CBF) via PR in a dose- and time-dependent manner, an effect reversible by estradiol or PR antagonists [56].

#### 3.3.4. Insulin and IGF-1 Receptors (IR, IGF1R)

Insulin receptors in type II pneumocytes regulate metabolism, alveolar homeostasis, and epithelial repair [57]. IGF1R, expressed on epithelial cells, fibroblasts, and endothelium, drives proliferation and remodeling after injury, but excessive activation promotes fibroblast-to-myofibroblast differentiation and fibrosis [57,58].

#### 3.3.5. Endothelin-1 Receptors (ETA, ETB)

ETA, expressed in vascular smooth muscle, mediates vasoconstriction and proliferation, while ETB, located in the endothelium, promotes nitric oxide and prostacyclin release and facilitates endothelin-1 (ET-1) clearance [59]. Overexpression of ET-1 and its receptors contributes to pulmonary hypertension, making them targets for selective or dual antagonists.

#### 3.3.6. Leptin Receptor (Ob-Rb)

Ob-Rb is expressed in bronchial epithelium, type II pneumocytes, and macrophages. It regulates alveolar homeostasis, immune activation, and macrophage phagocytosis, but can also amplify leukocyte infiltration and inflammation [60,61].

#### 3.3.7. Parathyroid Hormone-Related Protein (PTHrP) Receptor (PTH1R)

Secreted by type II pneumocytes, PTHrP acts on lipofibroblasts through PTH1R, driving lipid accumulation that provides substrates for surfactant synthesis. This epithelial–mesenchymal interaction stabilizes alveoli and prevents collapse [62,63].

#### 3.3.8. Vitamin D Receptor (VDR)

The VDR is expressed in epithelium and macrophages, where local 1a-hydroxylase activates 25(OH)D. VDR upregulates antimicrobial peptides such as cathelicidin and modulates inflammation [67,68].

### 3.4. Paracrine Pulmonary Activity

**Figure 3:**
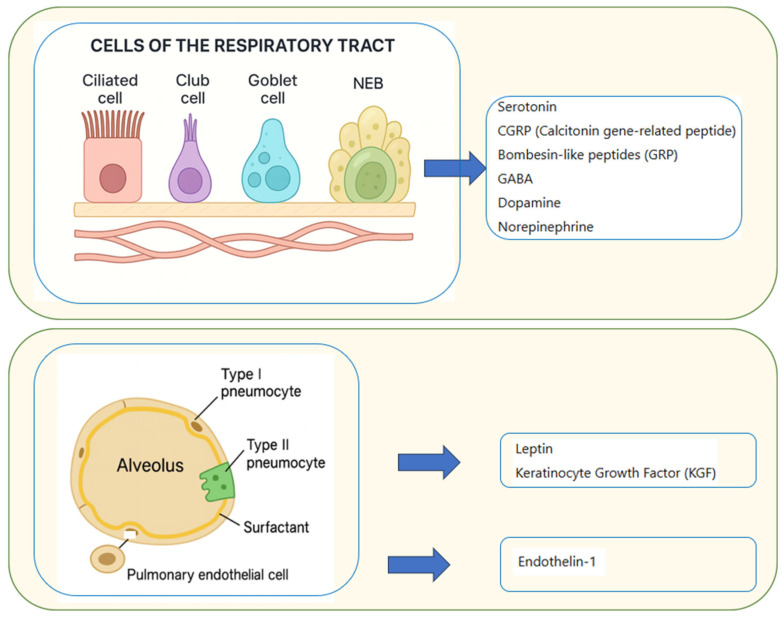
Schematic view of cells of the respiratory tract and alveolar epithelium function as active sources of endocrine and paracrine mediators. Pulmonary neuroendocrine cells (PNECs), a specialized subgroup of epithelial cells located mainly in the airway epithelium of large and small bronchi, occur either in isolation or as clusters termed neuroepithelial bodies (NEBs). These cells release serotonin, calcitonin gene-related peptide (CGRP), bombesin-like peptides (GRP), GABA, dopamine, and norepinephrine, thereby modulating ventilation, vascular tone, and immune responses [23,80,82,83]. In the alveolar compartment, type II pneumocytes secrete leptin and keratinocyte growth factor (KGF) [45,46], while pulmonary endothelial cells produce endothelin-1 [80], contributing to alveolar homeostasis, epithelial repair, and vascular regulation.

The pulmonary system is increasingly recognized as a dynamic endocrine and paracrine organ, producing mediators that influence local and systemic physiology. Key sources include PNECs, type II pneumocytes, and pulmonary endothelial cells, which together regulate vascular tone, immune responses, and epithelial homeostasis (Table 2; Figure 3).

#### 3.4.1. Pulmonary Neuroendocrine Cells (PNECs)

PNECs are a specialized epithelial subgroup, located mainly in large and small bronchi, either as solitary cells or clustered in neuroepithelial bodies (NEBs) [23,79] (Figure 3). They represent <0.5% of epithelial cells and are richly innervated by vagal and non-vagal afferents. Acting as hypoxia-sensitive chemoreceptors, PNECs release neurotransmitters (serotonin, GABA, dopamine, norepinephrine) and regulatory peptides (CGRP, GRP, somatostatin) [23,79]. These mediators modulate pulmonary blood flow (serotonin → vasoconstriction, CGRP → vasodilation), ventilation, and central nervous system (CNS)–lung communication. In addition, PNECs contribute to immune regulation and airway remodeling: their hyperplasia is characteristic of asthma, while CGRP and GABA actívate group 2 innate lymphoid cells (ILC2) and promote goblet cell differentiation [23,82,83].

#### 3.4.2. Pulmonary Endothelial Cells

Endothelial cells synthesize endothelin-1 (ET-1), a potent vasoconstrictor essential for pulmonary vascular tone and implicated in the pathogenesis of pulmonary hypertension [80,81].

#### 3.4.3. Type II Pneumocytes

Type II pneumocytes produce leptin and keratinocyte growth factor (KGF), which act in a paracrine fashion to maintain alveolar stability, stimulate epithelial proliferation, regulate surfactant, and promote tissue repair [63].

The secretion of these mediators is regulated by hypoxia, epithelial injury, inflammation, and mechanical stress, emphasizing the lung as an active sensor–effector unit at the crossroads of endocrine and immune regulation [84,85].

## 4. Endocrine Ailments with Pulmonary Involvement

### 4.1. Acromegaly

Acromegaly alters respiratory function due to excess GH and IGF-1, which cause macroglossia, pharyngeal hypertrophy, and craniofacial deformities, favoring upper airway obstruction and a >70% prevalence of obstructive sleep apnea (OSA) at diagnosis, sometimes with central apnea [86,87]. Functionally, there is an increase in lung volumes from thoracic expansion and possible alveolar hyperplasia [88,89], together with reduced carbon monoxide diffusion capacity (DLCO), suggesting early alveolocapillary damage [90]. Treatment improves these disturbances and can reduce OSA, although many patients still require continuous positive airway pressure (CPAP) [86,91].

### 4.2. Cushing’s Syndrome

Cushing’s syndrome markedly increases the risk of severe respiratory infections and pulmonary dysfunction due to combined immunosuppression, metabolic alterations, and respiratory myopathy. Chronic glucocorticoid excess leads to lymphopenia, neutrophil and monocyte dysfunction, and reduced complement activation, predisposing to bacterial, viral, and fungal infections, including severe COVID-19 [92,93,94]. Proximal myopathy compromises ventilatory muscles, favoring hypoventilation and dyspnea [92,95], while hyperglycemia and visceral obesity contribute to a restrictive pattern and reduced ventilatory reserve, overlapping with diabetes and obesity. Importantly, respiratory risk persists after remission due to lingering immunometabolic alterations [95,96].

### 4.3. Growth Hormone (GH) Deficiency

GH deficiency, especially with onset in childhood, is associated with delayed alveolization, reduced lung volumes, and respiratory muscle weakness, leading to decreased forced vital capacity (FVC), forced expiratory volume in 1 s (FEV_1_), and total lung capacity [97,98,99]. These deficits stem from reduced muscle mass and stature as well as direct effects of GH deficiency on lung development. GH therapy can partially improve respiratory function, especially if started early, although its long-term benefit remains under study [98,99]. Unlike other endocrinopathies, GH deficiency does not increase severe respiratory infection risk.

### 4.4. Thyroid Dysfunction

In hypothyroidism, hypoventilation occurs due to blunted ventilatory response to hypoxia/hypercapnia and mucopolysaccharide infiltration of the upper airway. DLCO is reduced even without obesity, and respiratory muscle strength decreases in proportion to TSH [14,100]. Levothyroxine replacement improves muscle function [90,101]. Up to 25% of severe cases present with small pleural effusions, usually resolving with treatment [102,103,104].

Hyperthyroidism causes dyspnea at rest and exertion via: (1) respiratory center hyperstimulation with increased CO_2_ sensitivity and ventilatory drive, (2) respiratory muscle weakness, especially diaphragmatic, and (3) reduced vital capacity and spirometric parameters (FVC, FEV_1_). These alterations are reversible with antithyroid therapy and restoration of euthyroidism [105,106,107,108,109,110].

### 4.5. Diabetes Mellitus

Diabetes produces a restrictive respiratory pattern with reduced FVC, FEV_1_, and DLCO [111,112,113,114], termed “diabetic lung”. Mechanisms include alveolar microangiopathy, collagen glycation, chronic low-grade inflammation, and autonomic neuropathy [115,116]. Impairment correlates with poor glycemic control and disease duration, showing an inverse relationship with spirometric indices [117,118]. Diabetes also increases susceptibility to severe respiratory infections [119], with evidence of a bidirectional relationship whereby reduced baseline lung function predisposes to diabetes [114].

### 4.6. Obesity

Obesity induces restrictive dysfunction, respiratory muscle weakness, and greater infection risk. Thoracoabdominal adiposity reduces lung compliance and diaphragmatic mobility, lowering expiratory reserve and functional residual capacity, and in severe cases, vital capacity and oxygenation [120,121]. Metabolically, adipokine-driven systemic inflammation (IL-6, C-reactive protein, CRP) promotes pulmonary dysfunction, pulmonary hypertension, and reduced exercise tolerance [122,123]. Fibro-adipogenic remodeling of the diaphragm further impairs muscle function [124,125]. Obesity also increases risk of severe infections (influenza, COVID-19) [126,127], and is linked to an asthma phenotype with bronchial hyperresponsiveness and systemic inflammation, as well as higher prevalence of OSA [128,129].

### 4.7. Hypoparathyroidism

Hypoparathyroidism, typically post-thyroidectomy or congenital, produces hypocalcemia that increases neuromuscular excitability and can trigger acute laryngospasm, with risk of airway obstruction [130]. Chronic hypocalcemia also causes respiratory muscle weakness and a restrictive pattern, while immune alterations modestly increase infection susceptibility [131,132].

## 5. Lung Conditions with Endocrine Manifestations

### 5.1. Non-Cancerous Lung Conditions

Chronic lung diseases not only generate respiratory damage, but also have a significant impact on endocrine homeostasis. Processes such as persistent inflammation, chronic hypoxemia and oxidative stress lead to alterations in metabolism, the gonadal axis, the GH/IGF-1 axis and bone health. Likewise, the bidirectional interaction between lung and endocrine system is enhanced by the treatments used, such as systemic corticosteroids, and by the frequent association with metabolic comorbidities.

#### 5.1.1. Chronic Obstructive Pulmonary Disease (COPD)

In COPD, systemic inflammation driven by tumor necrosis factor alpha (TNF-α), interleukin-6 (IL-6), and C-reactive protein (CRP) promotes insulin resistance, beta-cell dysfunction, and the development of type 2 diabetes and metabolic syndrome, independent of hypoxemia [19,133]. Hypoxemia, oxidative stress, and muscle loss contribute to hypogonadism and suppression of the GH/IGF-1 axis, partly due to reduced anabolic hormones and myokines regulating bone and gonadal metabolism [21,134,135]. Corticosteroid therapy and immobilization further accelerate osteoporosis, decreasing bone formation, increasing resorption, and exacerbating sarcopenia [136,137].

#### 5.1.2. Obstructive Sleep Apnea (OSA)

In OSA, intermittent hypoxia and sleep fragmentation activate the hypothalamic–pituitary–adrenal HPA axis, elevating nocturnal cortisol and increasing cardiometabolic risk [138]. CPAP reduces but does not always normalize cortisol [139]. OSA is also associated with male hypogonadism, with decreased testosterone from impaired GnRH and LH secretion; exogenous testosterone may worsen apnea by promoting pharyngeal relaxation [140,141]. A strong association exists between OSA and metabolic syndrome, with insulin resistance, dyslipidemia, and glucose dysregulation, driven by inflammation, oxidative stress, and endothelial dysfunction [22,138,142]. Although CPAP improves cardiovascular and some hormonal parameters, its impact on glycemic control and metabolic syndrome remains limited [142].

#### 5.1.3. Interstitial Lung Diseases and Chronic Hypoxemia States

Chronic hypoxemia, as in pulmonary fibrosis and pulmonary hypertension, alters bone remodeling, promoting osteopenia and osteoporosis. Hypoxia-induced reactive oxygen species (ROS) accumulation and hypoxia-inducible factor 1-alpha (HIF-1 a) activation inhibit osteoblasts, enhance osteoclastogenesis, and accelerate bone loss, compounded by inflammation and corticosteroid use [136,143]. This imbalance reduces bone mineral density and fracture threshold. In exacerbations of interstitial disease, euthyroid sick syndrome (low T3 with normal TSH) is frequent and serves as a marker of severity and poor prognosis [144].

### 5.2. Pulmonary Cancer/Neuroendocrine Tumors

#### 5.2.1. Pulmonary Neuroendocrine Tumors

The lung is a major site of neuroendocrine tumors, which range from low-grade typical and atypical carcinoids to highly aggressive small-cell lung carcinoma (SCLC) and large-cell neuroendocrine carcinoma (LCNEC). These tumors originate from PNECs and share the ability to produce peptide hormones and amines. Carcinoids usually present with localized disease and indolent growth, while SCLC and LCNEC display rapid progression, early metastasis, and poor prognosis [145,146,147].

#### 5.2.2. Pulmonary Endocrine Paraneoplastic Syndromes

Endocrine paraneoplastic syndromes are clinical manifestations resulting from ectopic secretion of hormones or peptides by malignant tumors, unrelated to tumor invasion. In the context of lung cancer, especially in SCLC, these syndromes are relatively frequent due to the neuroendocrine origin of the tumor. Their early identification may be key to both the diagnosis of the underlying tumor and the establishment of effective symptomatic treatment (Table 3).

Pulmonary NETs, particularly SCLC, are frequently associated with paraneoplastic endocrine syndromes due to ectopic hormone secretion. The most common is SIADH (syndrome of inappropriate antidiuretic hormone secretion), which causes hyponatremia, neurological symptoms, and worsened prognosis [148,149].

Another is ectopic adrenocorticotropic hormone (ACTH) syndrome, leading to Cushing’s syndrome with hypercortisolism, metabolic disturbances, and severe infections [150]. Less frequently, tumors may produce parathyroid hormone-related protein (PTHrP), causing humoral hypercalcemia of malignancy, which contributes to muscle weakness, arrhythmias, and decreased survival [151]. Bronchial carcinoid tumors, rarely (<1%) with liver metastases, may cause carcinoid syndrome characterized by flushing, diarrhea, and bronchospasm due to serotonin and other mediators. Diagnosis relies on elevated urinary 5-hydroxyindoleacetic acid (5-HIAA) and chromogranin A, and treatment with somatostatin analogues (e.g., octreotide) controls symptoms and hormone secretion [152].

These syndromes often precede tumor diagnosis, complicate management, and require combined treatment strategies, including oncologic therapy, hormone antagonists, and supportive measures to correct endocrine-metabolic disturbances.

## 6. Therapeutic Interferences

### 6.1. Inhaled Corticosteroids

Inhaled glucocorticoids in high doses can suppress the HPA axis, especially when combined with CYP3A4 inhibitors (such as ritonavir or ketoconazole), by increasing their bioavailability. Iatrogenic adrenal suppression manifests with fatigue, hypotension and adrenal crisis; it is recommended to monitor basal cortisol in chronic treatments and to use the minimum effective dose [153,154].

### 6.2. Endocrinopathies Induced by Immunotherapy

Immune checkpoint inhibitors (anti-CTLA-4, anti-PD-1, anti-PD-L1) are U.S. Food and Drug Administration (FDA)-approved agents for the treatment of multiple lung malignancies, including non-small cell lung cancer (NSCLC) and pulmonary melanoma, among others. Their mechanism is to potentiate the antitumor immune response, but this carries the risk of immune-mediated adverse events (irAEs), which can affect any organ, with endocrinopathies being especially autoimmune thyroiditis (initial hyperthyroidism followed by permanent hypothyroidism), hypophysitis (with ACTH or TSH deficiency) and, less frequently, primary adrenal insufficiency or autoimmune type 1 diabetes. The appearance of non-specific symptoms (fatigue, hypotension, polyuria) in patients treated with immunotherapy should prompt urgent hormonal evaluation [155,156,157,158].

### 6.3. Inhaled Insulin

Powdered human insulin formulations (Afrezza^®^) offer rapid postprandial control. Clinical trials have shown a small, reversible decrease in FEV_1_ and higher incidence of cough compared to subcutaneous insulin; therefore, they are contraindicated in patients with asthma or severe moderate COPD [159]. Baseline spirometry and annual follow-up is recommended to detect pulmonary deterioration [160].

## 7. Discussion and Future Directions

The integration of endocrinology and pulmonology offers new opportunities to improve mechanistic insight and clinical care. A central priority is the systematic characterization of the lung as an endocrine organ, including mapping of hormone production, receptor distribution, and paracrine signaling in the respiratory epithelium, vasculature, and immune niche. High-resolution single-cell and spatial transcriptomics will be key to defining these networks and discovering novel hormonal mediators of lung physiology.

At the translational level, endocrine phenotyping of patients with chronic lung diseases should be prioritized, focusing on alterations in the growth hormone/ insulin-like growth factor 1 (GH/IGF-1) axis, gonadal hormones, vitamin D metabolism, and adipokine signaling in COPD, interstitial lung disease, and obstructive sleep apnea. Large-scale longitudinal cohorts combining endocrine biomarkers, imaging, and respiratory function will help clarify causal pathways and prognostic value.

Therapeutically, hormone-based and hormone-modulating strategies represent promising interventions. Examples include thyroid hormone analogues or GH/IGF-1 modulation for lung repair and fibrosis prevention; selective estrogen or progesterone receptor modulators for asthma and airway hyperresponsiveness; and metabolic hormone mimetics (e.g., leptin or FGF21 analogues) for obesity-related respiratory dysfunction.

Finally, the intersection of immunoendocrinology and pulmonary medicine requires deeper study, particularly in chronic inflammation, immune checkpoint inhibitor–induced endocrinopathies, and respiratory infections such as coronavirus disease 2019 (COVID-19). Progress in this field will depend on interdisciplinary collaboration bridging molecular endocrinology, respiratory physiology, systems biology, and clinical trials. By advancing these lines, the endocrine–pulmonary axis may become a novel frontier for precision medicine in respiratory health.

This review has some limitations that must be acknowledged. It is a narrative rather than a systematic review, which may introduce selection bias despite the inclusion of the most relevant and up-to-date literature. Moreover, the available evidence on endocrine–pulmonary interactions is heterogeneous and frequently derived from experimental models or small observational cohorts, limiting the direct translation of findings into clinical practice. Several mechanistic insights remain hypothetical due to the scarcity of randomized trials or large prospective studies. Addressing these gaps through well-designed longitudinal cohorts, interventional trials, and integrative omics approaches will be essential to validate the proposed mechanisms, refine prognostic models, and develop targeted therapies at the interface of endocrinology and pulmonology.

## 8. Conclusions

The interplay between endocrine and pulmonary systems is bidirectional and clinically relevant, shaping pulmonary development, respiratory mechanics, immunity, and repair. Endocrine disorders such as hypothyroidism, hyperthyroidism, diabetes, obesity, acromegaly, Cushing’s syndrome, and hypoparathyroidism all produce characteristic respiratory manifestations, while chronic pulmonary diseases including COPD, OSA, and interstitial lung disease exert profound effects on endocrine axes such as GH/IGF-1, gonadal, vitamin D, and bone metabolism.

The concept of the “endocrine lung” highlights the local production of hormones and mediators by pulmonary cells, together with the expression of diverse hormone receptors in epithelium, endothelium, and smooth muscle, which enables direct hormonal regulation of pulmonary physiology and pathology.

Clinically, recognizing the endocrine–pulmonary axis opens opportunities for earlier diagnosis, risk stratification, and personalized interventions. Hormone-based or hormone-modulating therapies could become adjunctive strategies in respiratory medicine, complementing established treatments.

In summary, the integration of endocrinology and pulmonology provides a framework to better understand respiratory health and disease, emphasizing the need for interdisciplinary collaboration and translational research to translate these insights into precision medicine approaches.

## Figures and Tables

**Table 1:** Hormone receptors in the lung: localization, functions and clinical relevance.

Hormone/Mediator	Receptor	Pulmonary Localization	Main Functions	References
Thyroid hormones (T3/T4)	TRa, TRβ	Alveolar type II (AT2), regenerating epithelium	Alveologenesis, AT2→AT1 differentiation, antifibrotic	[48,49,50]
Glucocorticoids	GRa, GRβ	Epithelium, endothelium, airway smooth muscle	Anti-inflammatory, transcriptomic reprogramming, resistance (GRβ)	[51,52,53,54,55,56,57]
Estrogens	ERa, ERβ	Airway smooth muscle, bronchial epithelium	Bronchodilation, Ca^2+^ regulation	[58,59,60,61,62,63,64,65,66]
Progesterone	PR	Ciliated epithelium	Decreases ciliary beat (transcriptionally mediated)	[56]
Insulin	IR	AT2, airway epithelium	Alveolar fluid clearance, metabolic regulation	[57]
IGF-1	IGF1R	Epithelium, fibroblasts, endothelium	Cell proliferation, repair, fibrosis	[57,58]
Endothelin-1	ETA, ETB	Vascular smooth muscle (ETA), endothelium (ETB)	Vasoconstriction, vascular tone, remodeling	[59]
Leptin	Ob-Rb	AT2, bronchial epithelium, alveolar macrophages	Immune modulation, alveolar homeostasis	[60,61]
PTHrP	PTH1R	Lipofibroblasts (via AT2 secretion)	Surfactant synthesis, alveolar stability	[62,63]
Vitamin D (1,25(OH)_2_D_3_)	VDR	Epithelium, alveolar macrophages	Antimicrobial peptide induction, immune modulation	[67,68]

**Table 2:** Locally Produced Hormones in the lung.

Hormone/Mediator	Cell of Origin	Stimuli	Main Functions	Ref.
Serotonin	Pulmonary neuroendocrine cells (PNECs)	Hypoxia, neural stimulation	Pulmonary vasoconstriction, ventilation regulation, immune modulation	[23,79]
CGRP (Calcitonin gene-related peptide)	PNECs	Hypoxia, epithelial injury	Vasodilation, activation of ILC2, goblet cell differentiation, airway inflammation	[23,79]
Bombesin-like peptides (GRP)	PNECs	Hypoxia, neural inputs	Epithelial repair, immune modulation	[23,79]
Endothelin-1	Pulmonary endothelial cells	Shear stress, inflammation	Vasoconstriction, vascular tone regulation, pulmonary hypertension	[80,81]
Leptin	Type II pneumocytes	Inflammation, mechanical stress	Immune modulation, surfactant regulation, alveolar homeostasis	[73,74]
Keratinocyte growth factor (KGF)	Type II pneumocytes	Epithelial injury	Epithelial proliferation, tissue repair, surfactant regulation	[77,78]
GABA, dopamine, norepinephrine	PNECs	Hypoxia	Neurotransmission, ventilation regulation, CNS-lung communication	[23,79]

**Table 3:** Pulmonary endocrine paraneoplastic syndromes.

Syndrome	Associated Tumor	Prevalence	Clinical Features	Diagnostic Clues	Treatment	References
SIADH (Syndrome of Inappropriate Antidiuretic Hormone Secretion)	Small Cell Lung Carcinoma (SCLC)	~10–15%	Hyponatraemia, low serum osmolarity, high urine osmolarity, no edema	Unexplained hyponatraemia in smokers	Fluid restriction, hypertonic saline (if severe), vasopressin receptor antagonists (e.g., tolvaptan)	[148,149]
Ectopic Cushing’s Syndrome (ECS)	Mainly SCLC; also bronchial carcinoids	~1–5%	Rapid muscle weakness, hyperglycaemia, hypertension, hypokalaemia, metabolic alkalosis	High cortisol and ACTH; no suppression on high-dose dexamethasone	Ketoconazole, metyrapone, etomidate; oncologic therapy	[150]
Humoral Hypercalcemia of Malignancy (HHM)	Squamous cell carcinoma (NSCLC)	Less common than SIADH/ECS	Nausea, constipation, polyuria, confusion, coma (in severe cases)	Hypercalcaemia, low PTH, high PTHrP	IV hydration, bisphosphonates (e.g., zoledronic acid), denosumab (if refractory)	[151]
Carcinoid Syndrome	Bronchial carcinoid tumors (with liver metastases)	Rare (<1%)	Flushing, watery diarrhea, bronchospasm	Elevated 5-HIAA (urine), high chromogranin A	Somatostatin analogues (e.g., octreotide)	[152]
